# Sensory attributes of coffee beverages and their relation to price and package information: A case study of Colombian customers’ preferences

**DOI:** 10.1002/fsn3.1404

**Published:** 2020-01-16

**Authors:** Igor Barahona, Edis Mauricio Sanmiguel Jaimes, Jian‐Bo Yang

**Affiliations:** ^1^ Laboratory of Applications of the Mathematics Institute of Mathematics Universidad Nacional Autónoma de México Morelos México; ^2^ Enterprise and Regional Productivity Research Group (IPER) Faculty of Economics, Management and Accounting Sciences Universidad Libre, Campus Majavita Santander Colombia; ^3^ Alliance Manchester Business School The University of Manchester Manchester UK

**Keywords:** coffee beverages, label–package information, multivariate statistics, preference mapping, price, sensory analysis

## Abstract

Sensory analysis is a powerful tool for creating profiles of food and beverages based on information perceived by the human senses. This paper investigates 18 of the most popular Colombian coffees. Individuals from nine different cities assessed products in two different ways: degree of presence (absence) of sensory properties and degree of acceptance (liking). The results focused on identifying variations in sensory evaluations due to the city, as well as classification of the products according to their degree of acceptance or rejection, and investigating associations between sensory attributes, price, and label–package information. A correspondence analysis allowed us to investigate the variation introduced by the factor city. The most preferred/rejected products were identified through preference mapping. The level of intensity of the smelling sensory attribute positively affects the price and the information presented at the product´s label–package. However, tasting attributes negatively affects price and perceptions of the product´s label–package information. We conclude that smelling sensory attributes has greater impact on purchase intentions than tasting attributes. Decision‐makers should manage scent, price, and label–package characteristics wisely because they are part of the first experience of the customer.

## Graphical abstract

1) In this study, sensory attributes of the 18 most popular Colombian coffees are investigated. 2) Results suggest that sensory attributes linked to the olfactory sense have direct effect on price. 3) Attributes related to flavor have an inverse relation with price. 4) Same relations are observed for information showed at the package.

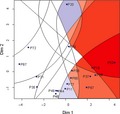

## 1 INTRODUCTION

Effective market segmentation is primarily based on having wide knowledge of the benefits that customers derive from products and services (Wedel & Kamakura, 2000). These benefits are directly related to attributes, which were previously revised by stakeholders (managers, designers, or researchers) during early pilot test stages (Armstrong & Kotler, 2013). In the case of Colombian coffee beverages, attributes are typically classified in three main groups: sensory, instrumental–functional, and expressive–symbolic (Wierenga, 1983). The first group is related to sensory properties of the beverages; the second refers to the nutritional properties and complementary aspects such as package size and shape. The third addresses the sense of social status, the notion of “being part of,” and what the customer wants to achieve by consuming the product.

A complementary criterion for classifying coffee beverages is determined by intrinsic and extrinsic signals. A signal is a mechanism that allows the consumer to perceive both the content and value of a given attribute (Blanco & Bello, 2002). Intrinsic signals gather those attributes that are part of the beverage and cannot be altered without changing its properties (Idoko, Nkamnebe, Ireneus, & Okoye, 2013). In the case of coffee, intrinsic signals refer to the sensory attributes of the beverage, such as the physical appearance, taste, and smell (Geel, Kinnear, & de Kock, 2005). Extrinsic attributes are factors that come from the environment surrounding the product. Price, label, personal values, and point of sale are examples of extrinsic attributes.

From the customer's perspective, it is not clear whether intrinsic values influence the extrinsic ones, or if there is an inverse order effect. There exists an active discussion in the literature, with evidence that supports both hypotheses. Theoretically, products can be conceived of as an array of product‐related cues. Each cue provides a basis for developing various impressions of the product itself (Dawar & Parker, 1994; Richardson, Dick, & Jain, 1994). Quality guidance, as introduced by Steenkamp and Van Trijp (1989) and Steenkamp and Van Trijp (1996), is an integrated consumer‐based approach that relates perceived quality judgments to physical product characteristics. In this approach, the first step requires identifying quality judgments; the second refers to disentangling the quality judgments from perceptions on intrinsic quality cues; and the third is translating consumer perceptions into physical product characteristics.

In the food and beverages consumer research field, sensory evaluations are of strategic importance for accurately assessing the satisfaction of customers (Babin & Griffin, 1998; Giese & Cote, 2000). Previous research has investigated associations among sensory attributes, as well as price and label information. Samoggia and Reidel (2018) provided evidence that the two most important factors influencing coffee purchase and consumption are sensory attributes and functional motives. Sepúlveda, Chekmam, Maza, and Mancilla (2016) affirmed that price influences purchase behavior as the type of coffee changes from traditional to social‐content. The likelihood of purchasing social‐content or organic coffee is lower than the intention to purchase traditional coffee. According to Van Loo et al. (2015), labels and packaging play an important role in communicating product characteristics (such as fair‐trade or social‐content), which later turn serve as drivers for purchasing decision. In this context, this work investigates to what extent sensory attributes—such as aroma, flavor, and fragrance—might be associated with the price that customers are willing to pay. Further, the association between information provided on the package and sensory properties of the product is also investigated. For instance, if a product with social‐content (fair‐trade) mentions outstanding sensory properties on the package label, then customers should be able to perceive those properties. Therefore, the current study has three objectives: to identify variations on sensory evaluations due to the city of residence of the participants, to classify products according to their degree of acceptance or rejection, and to investigate associations between sensory attributes, price, and label–package information. The paper is structured in five sections. A literature review is provided, followed by the materials and methods. Section four is reserved for the results. Discussion, limitations, and future lines of research are provided in section five.

## 2 LITERATURE REVIEW

According to Hoppert, Mai, Zahn, Hoffmann, and Rohm (2012), coffee beverage customers constantly demand more quality from sensory attributes. Therefore, it is of strategic importance for decision‐makers in the Colombian coffee industry to successfully evaluate customers’ preferences. As shown by Barahona, Sanmiguel, and Cavazos (2016) and Sanmiguel, Barahona, and Pérez‐Villarreal (2015), sensory analysis is a powerful tool that assesses to what extent some coffee beverages are preferred by customers. Pagès and Husson (2001) and Bécue‐Bertaut (2014) demonstrated that most of the existing research in the field focuses on three major lines: discrimination, typology, and consensus. The first attempts to respond to what extent beverages are either equal or different with respect to a given group. Typology research attempts to determine which attributes are most characteristic of the beverage. Consensus results when a group of tasters agrees on the presence (or absence) of certain attributes in a given beverage. To a lesser extent, researchers have also investigated sensory properties with extrinsic attributes. This is particularly true for the Colombian coffee industry, in which there is a need for sensory studies that support the decision‐making processes at the managerial level (Fermín, Soldevilla, García, & Bracho, 2012; Puerta, 2000). As Colombia is one of the main coffee producers in the world, investigating how the price and information printed on the package are related to sensory attributes is a novel contribution.

In Samoggia and Riedel (2018), a systematic peer‐reviewed journal search was performed. It included publications from Wed of Science, Scopus, and Sciencedirect, and covered a period from 2000 to 2018. From this search, a total of 115 articles that include the word “coffee” and at least one of the following words: “consumption,” “purchasing,” “attributes,” “behavior,” “perception,” and “willingness to pay,” were included in the study. The words should appear either in the title, topic, abstract, or keywords. Even though the number of published articles has dramatically increased in the last decade, this query yielded only one article related to coffee consumption in the Colombian context. This is indicative of the lack of attention that the Colombian market is receiving in the literature. Therefore, this work attempts to address this gap in the literature by contributing to the available studies that are focused on the Colombian context. Our study makes a novel contribution considering the low number of existing publications. Considering that sensory attributes of Colombian coffee are well appreciated on international markets (Sepúlveda et al., 2016), more research that allows a better understanding of this market is needed. We hope that managers, practitioners, and researchers in this country will find this work helpful, and that it will add value to their decision‐making processes.

Coffee is one the most preferred beverages worldwide (Moura et al., 2007). According to Di Donfrancesco, Gutierrez Guzman, and Chambers (2014), coffee is the second most consumed beverage in the world after water. The overall worldwide consumption of coffee in 2016 was estimated to be 155,470,000 sacks (60 kg each). The main consumers and nonproducing countries were those in the European Union, with approximately 42.6 million sacks in the same year. The second and the third nonproducing consumers were the United States and Japan, with 23.3 and 7.9 million sacks, respectively (ICO, 2017). Brazil led the world in producer country consumption in 2016, with 20.5 million sacks. Consumption in Brazil reached 4.90 kg/year/ inhabitant (Padovese, 2017). It was followed by Indonesia with 4.5 million and Ethiopia with 3.7 million sacks. Colombia, which is also a producer country, reached a consumption of 1.7 million sacks in 2016. The preceding represents an increase of 5% since 2013 (ICO, 2017). Most of the coffee consumed in Colombia is in the form of roasted and ground beans, which represents around 84% of the national market. The remaining 16% is brought to consumers as water‐soluble instant coffee (Euromonitor, 2017). During the last few years, there has been an increase in the presence of coffee capsules (pods) and it is expected this product will continue growing. Given a growing consumption of coffee beverages worldwide, it is evident that more sophisticated methods to better investigate consumer's preferences are needed. In this way, sensory analysis has been incorporated into the investigation of coffee consumers’ preferences.

Sensory studies were initially applied to the chemical analysis industry with the intent of characterizing food attributes. A peculiar property of sensory analysis is the fact that human senses are the measurement instrument. According to Lawless and Heymann (2010), sensory analysis is defined as groups of people who test samples of foods or beverages under controlled conditions, with the purpose of assessing attributes perceived by the human senses. Sensory studies are used in the food industry to characterize customer preferences. In the wine industry, sensory analysis is used to effectively investigate how people perceive those products. In the Colombian context, sensory analysis of coffee beverages was initially carried out by Feria‐Morales (2002) and Puerta (1997). Nowadays, official regulations, such SCAA (2016) and ICONTEC (2011), establish a certain minimum number of attributes that should be included in sensory studies to yield reliable results (see Table 1).

**Table 1:** Basic sensory attributes to be considered during an analysis

Attribute	Description
Fragrance	Sensation that is produced by volatile components of coffee when they are perceived by the nose without the addition of water. Fragrance is the smell of ground coffee without water.
Aroma	Corresponds to the olfactory perception produced by coffee's volatile compounds that are carried by water vapor (steam) at the time the beverage is prepared.
Acidity	Sensation produced by the acidic substances that are synthetized in hot water and later detected by the tongue. In general terms, acidity is determined by the growing region of the plant and with a lesser extent by toasting procedures.
Bitterness	Sensation produced by bitter substances present in coffee, which are extracted when hot water is added. They are especially perceived at the back the tongue.
Body	Strength, character, and heaviness of the drink associated to the characteristics of the raw material and the product–water ratio.
Sweetness	Basic gustatory sensation, mainly perceived at the tip of the tongue, which is associated with the presence of sweeteners.
Residual flavor	Post‐tasting flavors that remain in the mouth after tasting the beverage. They can be pleasant or unpleasant, depending on the initial characteristics of the raw material, processing and storage conditions.
Global Impression	Summarizes the panelists’ assessments regarding the overall impression of the tested sample.

Note

Own elaboration from: (Puerta, 1997; ICONTEC, 2011; SCAA, 2016).

According to Conti, Kitzberger, Scholz, and Prudencio (2013), coffee beverages can be classified into the following categories: traditional, premium, gourmet, and social‐content. Below is a brief description of each:

- *Traditional coffees*: Typically made by mixing two or more products and characterized for their low quality. They also correspond to the biggest and most popular market segmentation. In the context of the Colombian industry, their prices are lower than $3 USD per pound.
- *Gourmet coffees:* Commercial products that use raw materials such as premium coffee, defect‐free, and standard size grains. Their price might be between $4 and $6 USD per pound.
- *Premium or Excel coffees:* Produced under special conditions yielded by raw materials such as aromatic, fruity, and herbal smells. They are related to high quality in raw materials. The price for this segment might be above $7–8 USD per pound.
- *Social‐content coffees:* Products that are certified by international organizations such as Rainforest,^1^ Organic,^2^ UTZ,^3^ Fairtrade,^4^ among others. Their price is normally higher than the previous category, approximately $8 USD and higher.

In the context of sensory analysis, it is important to mention that the quality of coffee beverages is strongly influenced by the geographical and botanical origins of the grains, crop agronomic management, harvesting, washing and drying techniques, etc. Without considering geographical and botanical origins, all of these factors can be properly managed by professional producers (Aristizabal & Duque, 2006; Conti et al., 2013; Gimase, Thagana, Kirubi, Gichuru, & Kathurima, 2014; Puerta, 2013). To a lesser extent, there is a secondary group of factors that can be controlled to improve the quality, for instance, by storing, mixing, toasting, and grinding. Clarke (1987) found empirical evidence that the size of the particles has a strong influence on the process of preparing a coffee beverage with outstanding sensory characteristics. A third group is related to drink preparation techniques. The principle of preparation consists on the extraction of the soluble solids from the toasted and ground coffee by the addition of hot water. The main techniques used in Colombia to prepare the coffee beverages are as follows: pots, piston, percolator, fabric strainer, greca,^5^ drip, espresso, and pressured steam, among others (FEDECAFE, 2018).

On Manzo (2014) are proposed the “three waves of coffee consumption.” The first began in 1960s, and it was characterized by mass market with a generic commodity product. The second wave began in the 1990s with the emergence of coffeehouses, mainly Starbucks. Specialty coffee was introduced as response to a new type of consumer, who demanded more quality. The last wave began in the 2000s and it is characterized by the proliferation of small producers, who promoted specific methods for washing, drying, or roasting. Now, coffee can be considered a high‐value artisanal drink, similar to wine. From this, Sanmiguel et al. (2015) indicated that interest in coffee consumer behavior and sensory attributes has grown significantly during the last decade. In a study conducted by Van der Merwe and Maree (2016), it was found that people who like sensory properties of coffee also have positive attitudes toward specialty coffees. Similarly, young women are more predisposed to appreciate sensory properties than men.

Samoggia and Riedel (2018) affirmed that price attributes of coffee are widely studied in association with social‐content coffees and out‐of‐home consumption. Consumers are sensitive to changes in price. A decrease in the price yields an increase in purchasing habits. Similarly, Huang, Chang, Yeh, and Liao (2014) mentioned that the price promotions are positively correlated with sensory properties, service quality, consumer satisfaction, and further influence repeat purchase intentions. Andorfer and Leibe (2015) found that social‐content coffees are perceived as too expensive, but, at the same time, consumers might feel a moral obligation to purchase them. In Aguirre (2016), it was found that women are more sensitive to the price than men. On the other hand, men give more importance to the packaging than women. Van der Merwe and Maree (2016) found that men consume more specialty coffee than women.

Regarding to social‐content coffees, Van Loo et al. (2015) found that organic and fair‐trade are the two most important categories of social‐content coffees. Bissinger and Leufkens (2017) indicated that consumers prefer fair‐trade coffees over those with ecological or organic motivations. Loureiro and Lotade (2005) mentioned that packaging with information related to ethical or social‐content is an effective way to increase purchase intentions. In this form, consumers are willing to pay higher prices for social‐content coffees. Quintão, Brito, and Belk (2017) found that sensory skills expertize influences preferences. Consumers with higher sensory skills tend to prefer social‐content coffees. Cranfiel, Henson, Northey, and Masakure (2010) identified that variable price and package labels are the most important attributes affecting social‐content coffee consumption and purchasing behavior. Regardless of location, consumers are sensitive to changes on prices and different types of package labels. In the study conducted by Lange, Combris, Issanchou, and Schlich (2015), consumers were less willing to pay after they tasted social‐content labeled coffees. However, Lee, Bonn, and Cho (2015) found that consumers with a positive attitude toward organic coffee will pay higher prices because they belief it has health benefits.

In addition to the aforementioned characteristics influencing sensory attributes, a list of related factors—among them personal preferences, economic attributes, context of coffee consumption, and socio‐demographic—was proposed by Samoggia and Reidel (2018). In this context, we identified that most of the studies in the extant literature found that two factors, which are price and information provided at the package, have the highest impact on consumption behavior. Thus, we focused our attention on these two factors as is shown in the following sections.

## 3 MATERIALS AND METHODS

### 3.1 Participants

**Figure 1:**
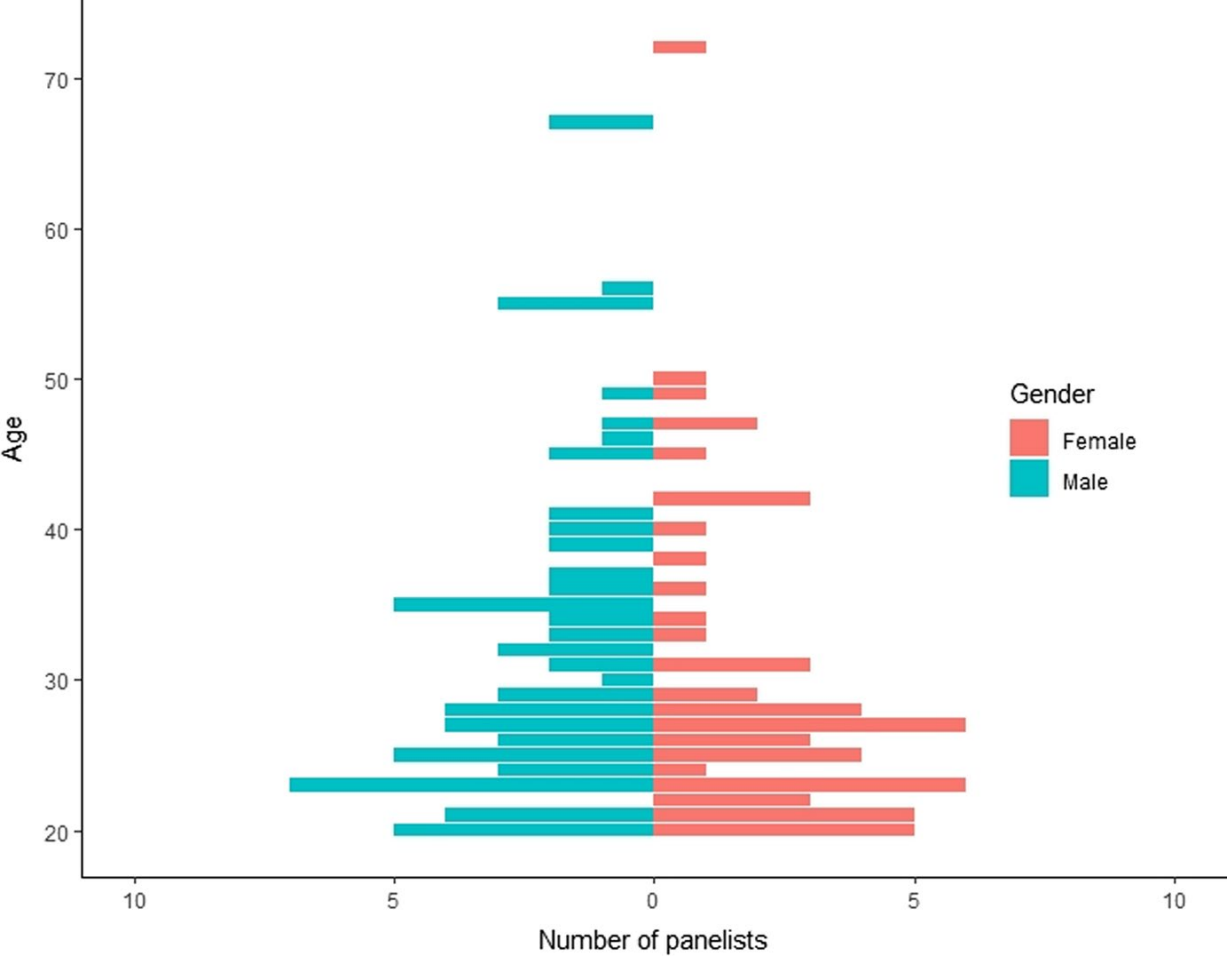
Distribution of variable age by gender

In total, 130 persons voluntarily participated in the study; 43% were women and 57% were men. The age average for women was equal to 29.9 ± 10.3 years and men reported an average age of 32.5 ± 10.7. Volunteers were recruited from nine different cities (see Table 2). While the age range for women was [20–72], men showed an age range of [20–67]. Distribution of variable age by gender is provided in Figure 1.

**Table 2:** Number of participants by city

City	Number of participants
Socorro	40
Charalá	30
San.Gil	28
Valle.de.San.Jose	12
Bucaramanga	8
Bogotá	4
Oiba	4
Barbosa	2
Duitama	2
Total	130

**Figure 2:**
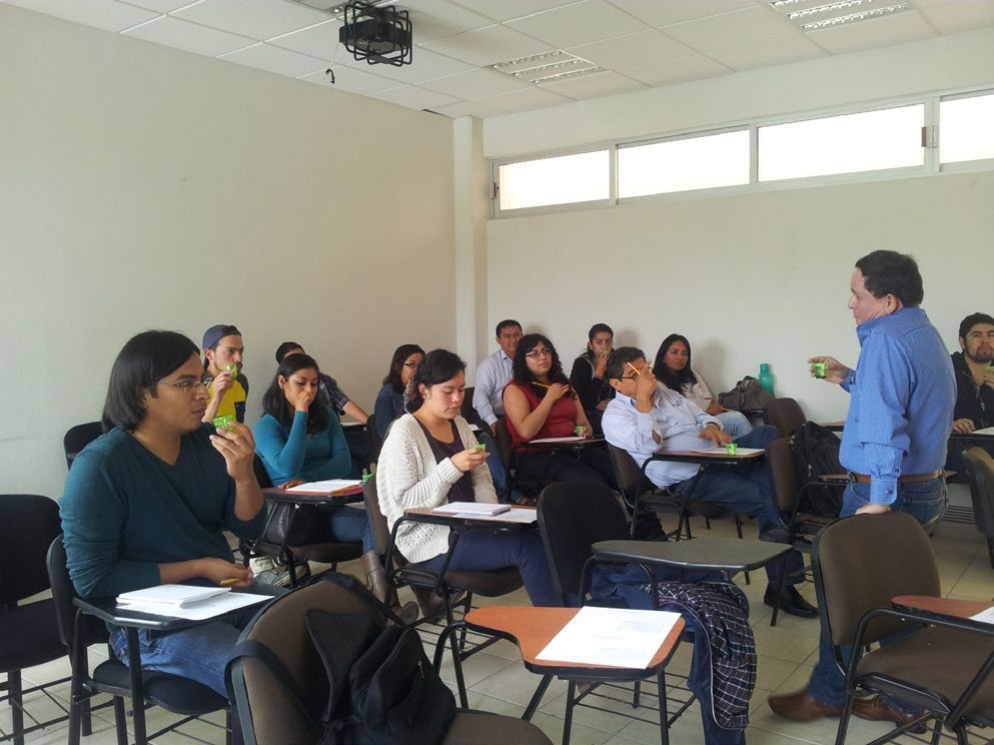
Illustration of how training was provided to panelists

Prior to the sensory evaluation of products, each group of volunteers received a 30‐min training in which sensory attributes of coffee beverages were explained. Volunteers were also instructed on how to use the evaluation sheet, which comprises 8 items in a Likert scale with 10 levels, in addition to demographic variables and codes for identifying panelists and products. While seven items referred to what extent sensory experiences are present in coffee beverages, the last item denotes the level of liking that the panelists gave to each product. A simulation was conducted, during which panelists practiced how a sensory evaluation session should be carried out. These results were dropped. We proceeded to the definitive assessment until all doubts were clarified. Figure 2 illustrates how the training was conducted for one group of panelists.

### 3.2 Data collection

From the wide range of ground and toasted coffee brands on the Colombian market, we selected the 18 brands that account for 84% of domestic consumption (Euromonitor, 2017). After conducting a detailed review of the information provided on the packages, each product was classified in one of four categories: traditional, gourmet, premium‐excel, and social‐content. The first category gathered coffees with prices lower than $3 USD per pound, which accounts for the majority of the domestic market in Colombia and is characterized by poor sensorial properties. Gourmet is in a price range of $4–6 USD per pound and mainly characterized by standard size in the coffee grains. The third category corresponds to premium coffees, which are typically produced under special conditions of weather, soil, and environment. Their prices are in a range of $7–8 USD per pound. Finally, social‐content coffees are typically certified by quality international organizations and they might have designation of origin. Prices for this category are about $8 USD per pound (see Table 3).

**Table 3:** Number of products for each category, according to product label information

Category	Number of products	Price range
Social‐content	3	$8 USD or lower
Premium	3	$7–8 USD
Gourmet	6	$4 to $6 USD
Traditional	6	$3 USD or lower

Considering that the prices varied slightly from one city to another, the average price of each product (including nine cities) was calculated. Participants agreed to assess at least two different products belonging to the same category. Each sensory evaluation was conducted by following the methodology suggested by FEDECAFE (2016), SCAA (2009), and ICONTEC (2011). The standardized process was as follows: A drip coffee maker was used, in which 5 grams of ground coffee was placed. Later, 10 ounces of water at 92° Celsius were gradually dripped through the coffee. Cups were labeled with a product code and filled with 20 milliliters each. Finally, these cups were distributed among the participants. The process was repeated as many times as the number of evaluated products. Regarding the testing space, each room was furnished with tables and chairs, on which panelists were randomly located. All participants were nontrained. Their occupations were students, freelancers, housewives, academics, and administrative employees. In accordance to the type of sensory technique applied, research shows that studies that use panelists without professional training might achieve results similar to those performed by expert tasters (Hayakawa et al., 2010; Lawless, 1984). This allowed us to minimize the bias due to different levels of experience and knowledge among the judges. The form in Appendix S1 was used for all evaluations.

### 3.3 Data analysis

At first, nonparametric statistical tests were conducted to find out whether there were significant differences between men and women across categories. The item referred to liking (overall impression) was used for this purpose. Later, a correspondence analysis, which is special kind of principal component analysis (PCA) for categorical data, was calculated to investigate relations between cities and sensory attributes. Associations between cities and liking were investigated. Then, a preference mapping, which is obtained by calculating the regression equation between the principal components and the liking score for each product, is presented. Finally, a structural equation model, based on the partial least squares (SEM‐PLS), is calculated to determine the two types of associations: How sensory attributes are related with price and the relation between sensory attributes and the information posted on the package. A detailed explanation of the statistical methods is provided in Appendix S1.

## 4 RESULTS

**Figure 3:**
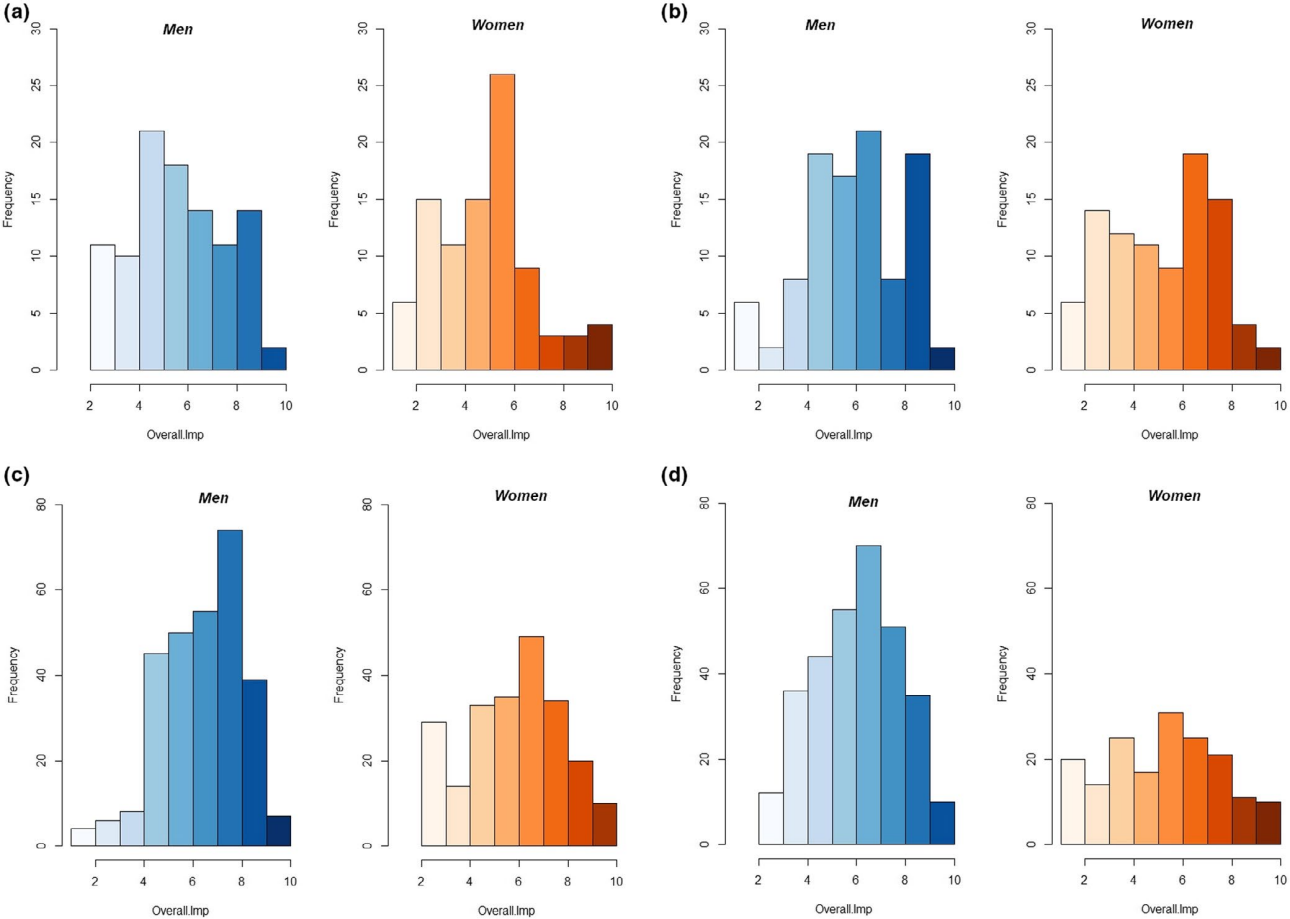
(a) overall impression for Gourmet. (b) Overall impression for social‐content. (c) Overall impression for premium. (d) Overall impression for traditional

From January 2016 to May 2018, people from Barbosa, Bogotá, Bucaramanga, Charalá, Duitama, Oiba, San Gil, Socorro, and Valle de San Jose agreed to participate in our research. A total of 130 participants assessed 18 different coffee beverages. All evaluated coffees were ground and toasted. The dataset under analysis contains a total of 1,386 observations and each is described by seven sensorial variables and one variable referred to liking. The above is in accordance with the criteria suggested by the National Federation of Colombian Coffee Producers (Cafeteros, 2019). The first analysis was focused on determining whether men perceive the beverages differently than women. Considering the item referred to product‐liking (overall impression), we performed tests for differences of means based on the Kruskal–Wallis statistical estimator—one test for each category. The average liking for gourmet coffees was *µ_f_* = 5.22 ± 2.07 and *µ_m_* = 6.07 ± 2.00 for women and men, respectively. For the social‐content products, they were equal to *µ_f_* = 5.58 ± 2.26 and *µ_m_* = 6.31 ± 2.10 for men and women (see Figure 3a,b).

Premium coffees reported an average liking equal to *µ_f_* = 6.27 ± 2.02 and *µ_m_* = 6.87 ± 1.67 for women and men, respectively. Finally, for traditional beverages these values were *µ_f_* = 5.67 ± 2.38 and *µ_m_* = 6.51 ± 1.77, for women and men (see Figure 3c,d).

Results for hypothesis tests are as follows, we failed to reject H_o_: There are significant differences among men and women cross the four categories: “gourmet” (χ^2^ = 7.55 and *p*‐value = .005), “social‐content” (χ^2^ = 4.81 and *p*‐value = .028), “premium” (χ^2^ = 11.10 and *p*‐value = .000), and “traditional” (χ^2^ = 14.35 and *p*‐value = .000). These hypotheses considered the liking (overall impression) as the input variable. From this perspective, we can argue that there are differences in how men and women prefer coffee beverages.

**Figure 4:**
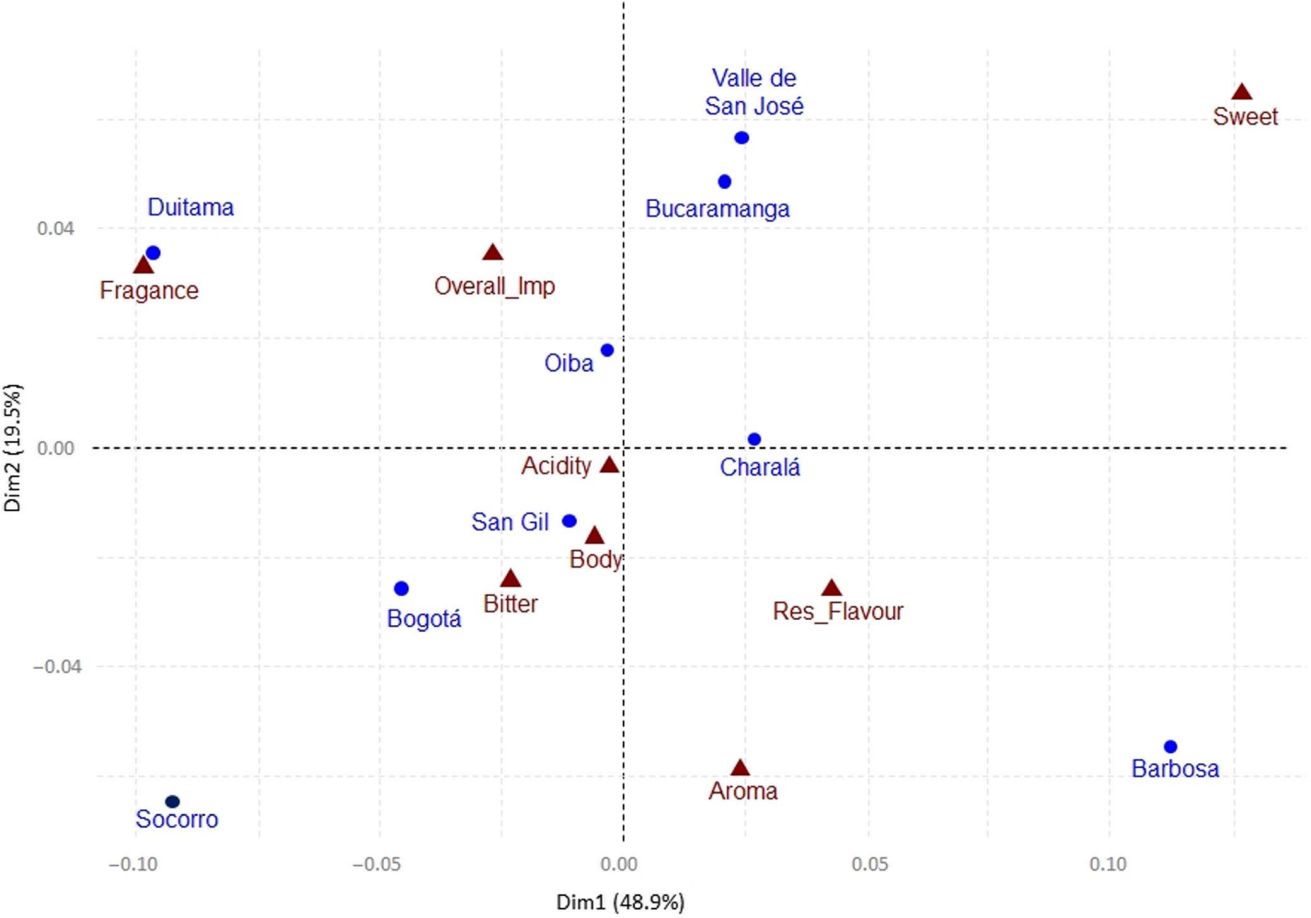
Correspondence analysis of sensory attributes and cities

The second part of the analysis consisted of identifying associations among cities and sensory attributes. Starting with a contingency table, a correspondence analysis (CA) was performed and the components with the biggest eigen values were used as axes to produce Figure 4. The eigen values (contributions) of eight items, of which seven referred to sensory attributes and one related to product‐liking, were interpreted as Euclidian distance. The categorical variable city with nine levels was also interpreted in this way. The above allowed us to visually represent the mentioned variables on the factorial plane. For instance, San Gil is closer to “Body,” “Acidity,” and “Bitter”; similarly, Duitama is closer to “Fragrance.” Conversely, two cities, Socorro and Barbosa, are further from the sensory attributes. We conclude that panelists from the last‐mentioned cities show no preference for any sensory attribute, in contrast to Bogota, which is closer to “Bitter.” In this form, Figure 4 provides an easy and intuitive way for representing the relationships between the investigated cities and sensory attributes and liking.

**Figure 5:**
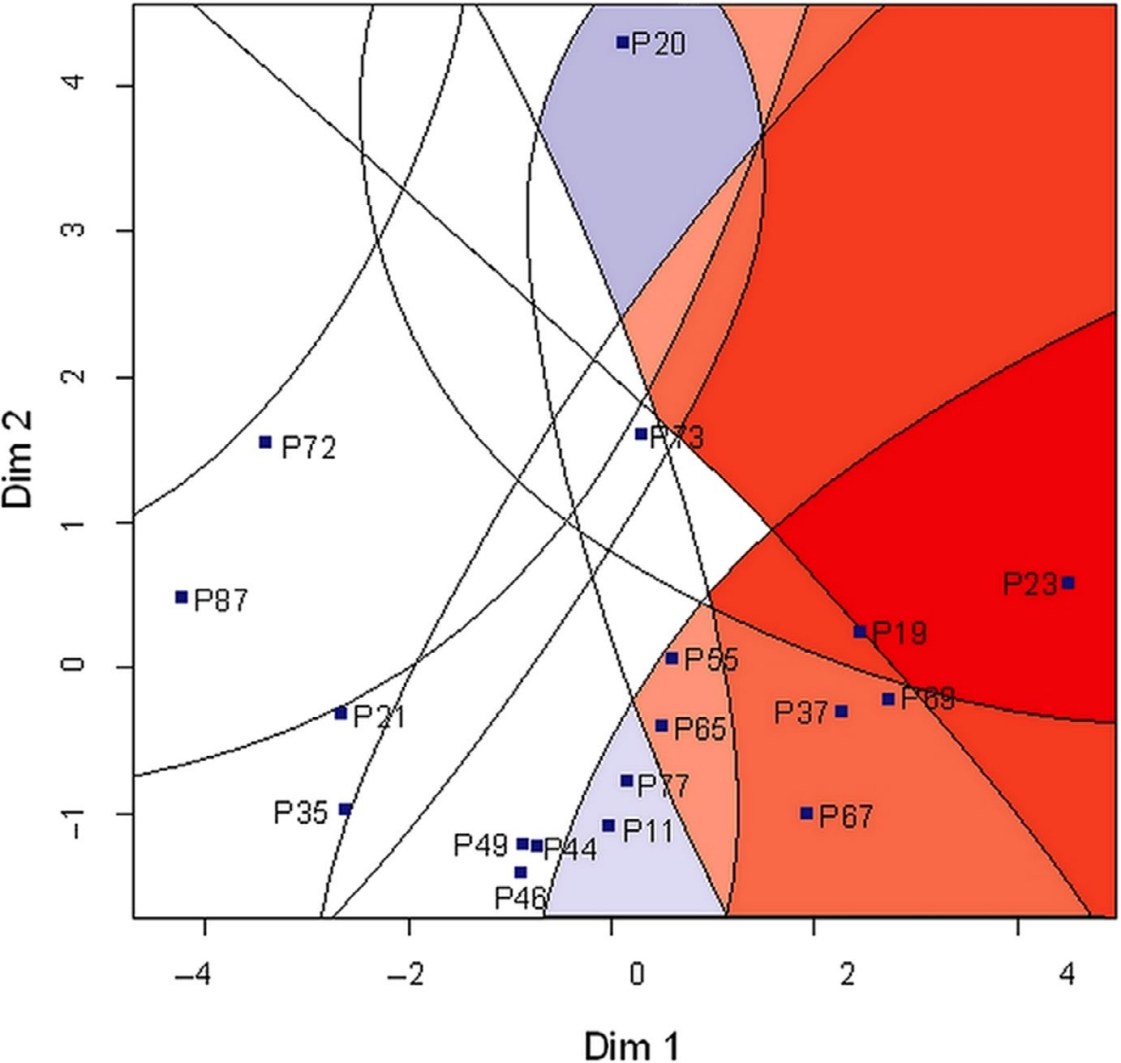
Internal preference mapping

It is of interest which beverages are most preferred. With this regard, preference mapping (PM) is a powerful tool to reach a deeper understanding of customer tastes. Here, it is illustrated how PM is used to assist managers and developers in the Colombian coffee industry, so as to identify the mixture of beverages that maximize customer preferences. Decision‐makers can mine PMs for useful information for successfully carrying on brand positioning and segmentation. On the basis of 18 different coffee beverages, a PM was prepared and presented as Figure 5. While several shades of red indicate different levels of liking, blue and white colors denote levels of dislike. The stronger the shade, the higher the levels of like or dislike. This PM was prepared with the variable of overall impression as input.

According to Figure 5, the most preferred beverage was P23, followed by P19, P69, P37, and P67. The products that showed the least acceptance were P20, P11, and P77. Products that were moderately accepted by panelists on white areas of the PM are represented. For instance, P87, P72, P35, and P21 are considered “neutral,” as they were neither accepted nor rejected. These products can be also deemed “grey” beverages because they do not stand out as positive or negative with their sensory properties. PM is a useful tool to differentiate levels of preference and rejection concerning a group of products and to later visualize this information on an intuitive form. Considering the variable “global impression,” statistical tests were conducted to find out whether there were significant differences across products. No significant differences were found on thirteen pair of products, which represent the 4% of the total possible pairs (18 × 18–18). Averages for each product, with respect to the sensory assessment, are provided in Table 4.

**Table 4:** Averages obtained for each product with respect to the sensory attributes

Product	Aroma	Acidity	Fragrance	Bitter	Body	Sweet	Residual flavor	Overall impression
p11	6.2	5.1	6.9	6.0	5.6	4.8	5.7	6.3
p19	6.4	6.2	6.8	7.0	6.4	5.0	6.6	6.8
p20	4.8	7.6	6.0	7.7	6.2	3.9	6.7	4.8
p21	5.3	4.2	6.1	5.6	5.5	3.6	5.3	5.8
p23	6.3	6.9	7.8	7.3	6.8	5.1	7.3	7.3
p35	5.5	4.9	6.1	5.0	5.5	3.5	5.0	6.2
p37	6.3	6.4	6.6	6.4	6.5	5.6	6.4	7.0
p44	5.8	5.3	6.9	5.1	5.6	4.0	5.8	6.5
p46	5.8	5.4	6.2	5.1	5.3	5.5	5.7	6.2
p49	5.6	5.2	6.6	5.2	5.5	4.7	5.9	6.3
p55	5.7	5.6	6.7	6.4	6.1	5.1	6.0	6.2
p65	6.2	5.7	6.4	6.1	6.0	4.3	6.0	6.6
p67	5.9	5.5	7.5	6.0	6.6	5.6	6.1	6.7
p69	6.5	5.9	7.2	6.6	6.6	5.0	6.9	6.8
p72	4.8	5.1	6.7	5.9	5.4	2.5	5.0	4.5
p73	5.5	6.3	6.5	7.0	6.1	4.0	6.0	5.9
p77	5.9	4.9	6.8	5.9	6.1	4.4	6.0	6.5
p87	4.4	4.8	6.2	5.5	5.0	3.1	4.6	5.2

The last part of this section focuses on investigating two types of effects. First, the effect of sensory attributes on price, and, second, the impact of the same sensory features on the information printed on the package. The key idea is to figure out how the sensory properties of coffee beverages might influence customer perception related to price and information printed on the package. Prior the preparation of the structural model, sensory attributes were clustered in two groups. The first was related to olfactory properties, and the second grouped tasting characteristics.

**Figure 6:**
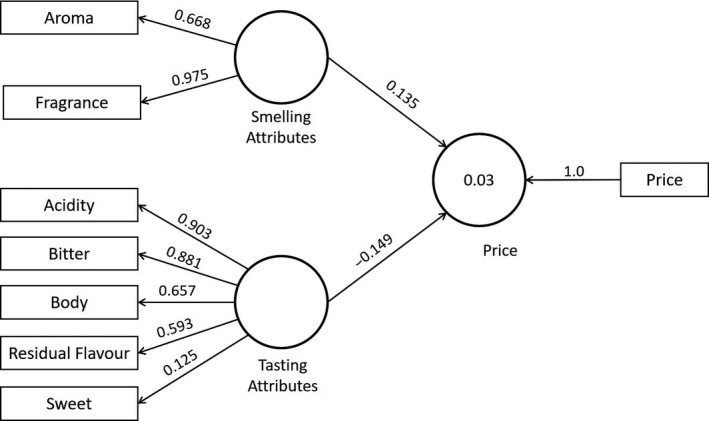
Structural equation model for sensory attributes and price

A diagram for the structural equation model on Figure 6 is provided. It illustrates the relation between sensory attributes and price. The model is composed of seven indicators separated into two latent variables: smelling and testing properties. With exception of “sweet,” all loadings for indicators were higher than 0.40. The “fragrance” obtained the highest loading, equal to 0.975, followed by “acidity” and “bitter,” which showed loadings of 0.903 and 0.881, respectively. Considering our data are not normally distributed, it is not appropriate the use of the maximum likelihood estimators to conduct hypothesis testing related to the significance of factor loadings and path coefficients. As an alternative course of action, we apply the bootstrapping procedure proposed by Hair, Hult, Ringle, and Sarstedt (2016). In this form, a total of 500 samples were generated from the original data with replacement. Using these data, we calculated χ^2^ (chi‐squared) statistics estimators for all elements of the model. We tested H_o_: The element of the model (sensory indicator or path coefficient) is different to zero with a threshold equal to 1.96 and significance of 95% (*α* = 0.05; two‐tailed test). Excepting the “sweet” attribute, we failed to reject H_o_ for all elements of the model. In this case, the Normed Fit Index (NFI) was equal to 0.651 (χ^2^ = 991.52), and the Standardized Root Mean Square Residual (SRMR) was 0.114. Considering the loading for the sensory attribute “sweet” was lower than 0.40, we calculated an alternative model by removing this attribute. After this, measures for goodness of fit were again calculated for a second model, with values equal to NFI = 0.702 (χ^2^ = 766.32) and SRMR = 0.097.

The effect of the latent variable “smelling attributes” on price was equal to 0.135. This path coefficient can be interpreted as follows: A change in smelling attributes by one standard deviation from its mean will increase by 0.135 the standard deviation of the variable price from its own mean. On the other hand, the latent variable “testing attributes” reported a negative effect on price equal to −0.149. According to Hair, Sarstedt, Ringle, and Mena (2012), a negative path coefficient implies an inverse relation among the studied variables. In this case, an increase by one standard deviation from its mean on testing attributes will cause a decrease by −0.149 on price from its own mean.

**Figure 7:**
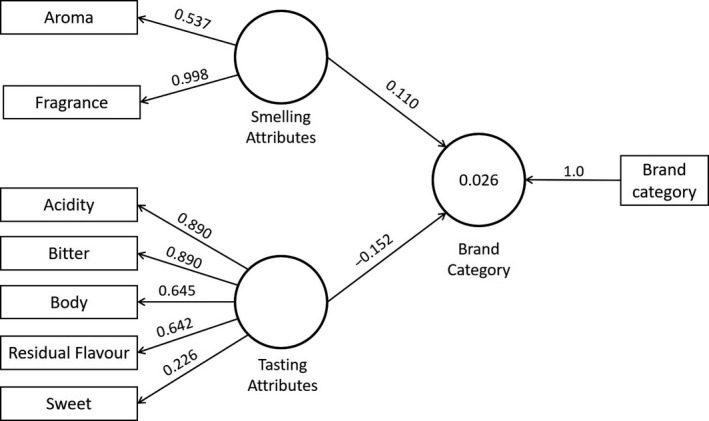
Structural equation model for sensory attributes and brand category

The structural equation model, which illustrates the relation of sensory attributes and the information printed on the package, is presented in Figure 7. In this case, it used the previously introduced classification of the seven sensory indicators. Excepting the “sweet” indicator, all loadings were higher than 0.40. The highest loading was for “fragrance,” followed by “acidity” and “bitter,” which were equal to 0.994, 0.890, and 0.890, respectively. The measures of goodness of fit were as follows: NIF = 0.700 (χ^2^ = 862.33) and SRMR = 0.110. By removing the attribute “sweet” because of its low loading, a second model was estimated. In this last case, we obtained measures of goodness of fit equal to NIF = 0.742 (χ^2^ = 663.06) and SRMR = 0.102.

A bootstrapping with 500 samples without replacement was carried out to assess the significance of all elements of the model. We tested H_o_: The element of the model (sensory indicator or path coefficient) is different to zero, and we failed to reject the null hypothesis in all cases, except for the “sweet” indicator. The above with a threshold of 1.96 and significance equal to 95% (*α* = 0.05; two‐tailed test). According to Figure 7, the effect of “smelling attributes” on information printed on the package was equal to 0.110. This means that changing smell properties by one standard deviation from its mean will increase by 0.110 the standard deviation of the indicator related to the information provided at the package, from its own mean. Besides, an inverse relation was observed between the construct testing properties and the same product package information. The negative path coefficient represents an increase on the latent variable testing attributes by one standard deviation from its mean, which will cause a decrease by −0.152 on the standard deviation of product package information, from its own mean.

## 5 DISCUSSION

In this paper, our findings were structured in four parts. First, we investigated whether women and men differently perceive coffee beverages across the four investigated categories. Our results are consistent with Van der Merwe and Maree (2016), who found differences by gender, in the sense that men prefer social‐content coffee more than women. Considering gender as socio‐demographic variable, these results contribute to the discussion of differences in coffee preferences given by the gender, as it is reported by Samoggia and Riedel (2018). Similarly, Spence and Wan (2015) consider drinking coffee a multidimensional experience that involves factors as sensory properties of the beverage, price, label–package, context of the consumption, and socio‐demographics, among others. Therefore, this contributes to the discussion of how these factors influence consumer preferences, with special interest on price and label–package.

Subsequently, a correspondence analysis allowed us to identify the extent to which consumers from nine Colombian cities perceive coffee sensory attributes differently. Significant differences were found with respect to the investigated cities. For instance, Duitama is closest to “fragrance,” Bogotá is closest to “bitter,” and San Gil is closest to “body.” These results provide additional evidence that the variable city introduces variability to the form in which sensory attributes are perceived. There are several works that investigate sensory attributes in Colombian coffees, among them Di Donfrancesco et al. (2014), Sanchez and Chambers (2015), and Sanmiguel et al. (2015). However, to our knowledge, this work is the first study that investigates differences on how coffees are perceived with respect to Colombian cities. In this form, we make a modest contribution by providing empirical evidence that contributes to the discussion about how the variable city influences sensory testing.

Having studied the differences influenced by gender and city, we find it necessary to focus our attention on variations due to different beverages. A preference mapping (PM) was used to identify which products were among the most preferred or rejected. While products P23 and P19 were the most preferred, products P20, P11, and P77 were the most rejected. Using PM for discriminating products based on their degree of acceptance is not a new idea. According to Perrot et al. (2018), in the last 30 years more than 270 papers have been published using the phrase “Preference mapping” either in the title, abstract, or keywords. A subset of 223 papers refer to empirical studies: 73 for fruits and juices, 58 for meat and fish, 54 for processed foods, and 38 for nonfood consumer goods (e.g., tobacco, cosmetics, cars, telephones, printers, etc.). From the previous list, only four published articles focused on analyzing the sensory properties of coffee, and two of those focused on Colombian coffee. From this perspective, we consider the sensory analysis of coffee to be an emerging discipline and that Latin American countries should be included in this research because they are important coffee producers. On the other hand, Bonany et al. (2014) investigated consumers’ preferences across 7 European countries and tested 11 apple varieties, by using PM in a similar approach as was used here to assess coffee beverages. The work by Geel et al. (2005) is among the first studies focused on using PM for investigating coffee beverages. We believe decision‐makers, in the context of the Colombian coffee industry, might find these results helpful for improving their process and products.

Structural equation modeling (*SEM*) is used to describe the correlational structure among observed and latent variables. In the framework of sensory analysis, several studies have demonstrated the suitability of *SEM* for testing theoretical prepositions referring to how latent variables are linked to empirical data and the direction of that relationship (Abdi, Valentin, Chollet, & Chrea, 2007; Alonso, Gallego, & Mangin, 2005; Martínez‐Carrasco, Brugarolas, Martínez‐Poveda, Ruiz, & García‐Martínez, 2012; Vilela, Marques, & Correia, 2018). According to Hair et al. (2016), the *SEM* approach comprises two types of models: measurement and structural. While the first defines how latent variables are operationalized in terms of the observed variables, the last describes relations among latent variables. Our model, which refers to the relationship between sensory attributes and price, registered factor loadings higher to the threshold suggested in the literature, equal to 0.40 (Chin, 1998; Hair et al., 2016, 2012; Hair, Ringle, & Sarstedt, 2013) for all cases except for the “sweet” attribute. The same pattern was observed on the model that describes the relations among sensory attributes and information printed on the package. In this form, latent variables are represented in Figures 6 and 7 are properly characterized by the observed data.

Subsequently, all path coefficients for both structural models were significantly different to zero (price and information printed on the package). It brings attention to the fact that the path coefficient for “smelling attributes” was positive, which means that more intensive smelling properties are associated to higher prices. In the study conducted by Nadiri and Gunay (2013), the smell of coffee in the shops of a multinational chain was associated with customers’ positive experiences and higher purchase intentions. Labbe, Ferrage, Rytz, Pace, and Martin (2015) found that consumers who drink coffee for sensory pleasure give more importance to coffee smell, followed by the taste. They are also willing to pay higher prices to obtain that sensory pleasure. On the other hand, the negative path coefficient among testing attributes and price is explained by the fact that customers are not willing to enhance their purchase intentions due to the increase in quality of testing attributes. Winchester, Arding, and Nenycz‐Thiel (2015) found that price is the primary reason why consumers do not purchase social‐content coffees because they find them “too expensive.” Samoggia and Reidel (2018) mentioned that consumers in general are sensitive to variations on price. Products with higher quality on tasting sensory properties are associated to higher prices and thus, the likelihood of purchasing them is lower.

Finally, we discuss the relation of both smelling and testing attributes and the information printed on the package. In the first case, a positive path coefficient indicates that smelling attributes create a positive perception of the information printed on the package. Customers associate more positive (pleasant) smells of coffee to products with packages labeled as social‐content, which also are more expensive. In Lund (2015), a literature review was conducted, which demonstrated that smelling elicits a positive influence in different context and scenarios, such as a larger duration of visits in shops, higher customer satisfaction on surveys, and improved perception of product quality. In this case, evidence supports that smelling is positively associated with the label–package information, which is also coherent with results reported on other works (Krishna, Elder, & Caldara, 2010). However, tasting attributes are negatively associated with the label–package. Consumers believe that social‐content coffees taste worse than traditional ones. In Obermiller, Burke, Talbott, and Green (2009), similar results were reported in the sense that consumers associate more unpleasant flavors to social‐content coffees (fair‐trade). Taste was identified as the second most important barrier for purchasing social‐content coffees.

In most cases, the smelling sensory property is the first variable with which consumers make contact. If this occurs when the package is still closed, then consumers create a first impression of the product sensory properties based only on smelling, which leaves the tasting attributes to second place. Hence, smelling attributes have larger impact on purchase intention than tasting attributes. Managers, marketing specialists, coffee producers, and decision‐makers should manage smelling and label–package characteristics wisely because they are part of the first experience of the customer. Considering the positive correlation between olfactory attributes and price, we suggest that decision‐makers consider this link when launching a pricing campaign, as pleasant aromas might lead to higher prices and therefore more benefits for sellers. As is it stated by Li, Streletskaya, and Gómez (2019), sensory variables are always related to extrinsic aspects of the product and they influence the preference level and purchase intention. This work intends to provide basic guidelines about how sensory attributes should be prioritized to maximize benefits for customers and producers.

With the small sample size, our results are limited and it is not possible to generalize these findings to the Colombian population. Our contribution is illustrative rather than conclusive. The motivation is to exemplify how quantitative methods can produce novel knowledge about coffee products, rather than to provide overwhelming evidence in favor of the conclusions here presented. Future studies should validate the robustness of our results by using a larger sample, with more participants per city. Future research should also assess the validity of these conclusions across Latin American countries, which share socio‐demographic similarities and might lead to similar results.

## CONFLICT OF INTEREST

The authors declare that they do not have any conflict of interest.

## ETHICAL APPROVAL

This study does not involve any human or animal testing.

## INFORMED CONSENT

Written informed consent was obtained from all study participants.

## ENDNOTES

1
https://www.rainforest-alliance.org/

2
https://eocc.nu/members/bcs-oko-garantie/

3
https://utz.org/

4
https://www.fairtrade.net/

5 Equipment to prepare infusions of coffee used especially in public places.
