# Supplementary material for: Sensory attributes of coffee beverages and their relation to price and package information: A case study of Colombian customers’ preferences
Source: Food Sci Nutr. 2020 Jan 16;8(2):1173–86. doi: 10.1002/fsn3.1404 (PMC7020298; doi:10.1002/fsn3.1404)
Supplement: Supplementary file 1 [file FSN3-8-1173-s001.docx]

**Appendix A.** Evaluation form for sensory analysis of coffee

**Appendix B. Statistical Methods**

Principal Components Analysis (PCA) is a well-known method for tackling data composed of multiple variables. The starting point of a PCA is a matrix **X**=[x_1_,x_2_,…,x_p_], on which *p* variables are collected for *n* individuals, and thus **X** is of order *n* x *p*. Principal components as uncorrelated variables, which are able to explain most of the variability of **X**. A remarkable property of PCA is the capacity of creating intuitive “easy to read” visualizations, taking as starting point a reduced dimensional space. Under this framework, the Euclidean distance among two rows $x_{i}^{'}=\left( x_{i1},x_{i2},\ldots,x_{ip} \right)$ and $x_{j}^{'}=\left( x_{j1},x_{j2},\ldots,x_{jp} \right)$ is given by the following formula:

$\delta_{ij}^{2}=\left( x_{i}-x_{j} \right)^{'}\left( x_{i}-x_{j} \right)=\sum_{h=1}^{p} \left( x_{ih}-x_{jh} \right)^{2}$ (1)

On formulation (1), $\delta_{ij}^{2}$ is a squared matrix with dimensions *n* x *n* that contains the distances among individuals. Consequently, we can represent *n* rows of **X** as equal number of points on $\mathbf{R}^{n}$. Note that the Principal Components (PC) are obtained from the correlation matrix, on which the covariance of each observed variable with respect to itself is equal to 1. Therefore, a PC with a value lower than 1 explains less variability than the original variable. We are retaining the first *m* components that satisfy $\lambda_{m}$ ≥ 1, where $\lambda_{1}\geq\cdot\cdot\cdot$ ≥ $\lambda_{p}$ are the eigenvalues of the correlation matrix **R**.

Later, the two PCs with the biggest eigenvalues are used to generate maps of preference. Preference Mapping (PM) is a multidimensional representation of products, given their sensory properties such as colour, flavour, or aroma. The literature distinguishes two types. While internal maps are PCAs applied to acceptance data; external maps related to acceptance test data with intensity of sensory attributes. The Danzart method of calculating a quadratic regression among the fists two PCs in one hand, and the scores provided by customers on the other. Below is its mathematical representation.

(2)

$$TP_{n}=\alpha+\sum_{i=1}^{n} \beta_{i}X_{i}+\sum_{i=1}^{n} \delta_{i}X_{i}^{2}+\sum_{i=1}^{n} \sum_{i=1}^{n} \gamma_{ij}X_{i}X_{j}$$

In formulation (2), *TP*_n_ represents the total preference of customer *n* on the product *i*. Besides, the product score on PC*_i_* is represented by **X***_i_*. The regression coefficients of the model are denoted by α, β and δ. Note that (2) is a quadratic equation and therefore our preference map is composed of parabolic lines. The principal components with the biggest eigenvalues are used for generating two-dimensional visualizations. The number of degrees of freedom in the regression is given by the total number of observations minus one.

The Partial-Least Squares – Structural Equation Modelling (PLS-SEM) is aimed to establish casual relationships among two or more variables by maximizing the explained variance of latent constructs under analysis. This is one of the main differences from those methods based on the covariance matrix (CB-SEM), which are focused on reproducing a theoretical covariance matrix. Both families of methods are composed of three parts: a structural model, measures of the model, and weights. Both families of models also consist of two types of variables: measurement and latent variables. The latter group can be also divided in exogenous and endogenous. If the latent variable does not have any predecessor, it is considered exogenous, otherwise it is endogenous. The mathematical notation for the general case of one PLS-SEM model can be expressed in the following way.

$Y^{'}=\boldsymbol{YB}+\varepsilon$ (3)

In formula (3), ***Y*** denotes the matrix of latent variables, ***B*** is reserved for the matrix coefficients and ε represents the random error associated to the model. For centred models we have E(ε)=0. In addition, each element of matrix ***B***, where ***i****=*1*,…,n* and ***j****=*1*,…,m* is equal to *b_i_*_,_*_j_*=0, for those elements of the adjacency matrix ***D*** which are also equal to zero. Measurement Variables (MV), which are indeed the observed ones, relate to the outer part of the model. The group of MVs, which are related to a unique latent variable, is named block (*g*). In this way, one PLS-SEM model is composed as many groups as the number of latent variables. Prior the construction of the model, all MVs should be scaled with parameters µ=0 and σ=1. The mathematical (reflective) relation among latent variables and MVs, for the block *g*, on formulation (4) is shown.

$X_{g}=y_{g}w_{g}^{T}+F_{g}$ (4)

On (4) $w_{g}^{T}$ represents the weights of the multivariate regression, which are estimated through partial least squares algorithm. Moreover, $E[F_{g}\mid$ $y_{g}]=0.$ In this form, for the block $\boldsymbol{X}_{g}$ , which correspond to the latent variable $y_{g}$ is measured by **X**_1_,…,**X***_n_* manifest variables with their respective ***w***_1_,…,***w****_n_* individual weights.
